# A comparative study of respiratory syncytial virus (RSV) prophylaxis in premature infants within the Canadian Registry of Palivizumab (CARESS)

**DOI:** 10.1007/s10096-012-1617-7

**Published:** 2012-05-01

**Authors:** B. Paes, I. Mitchell, A. Li, K. L. Lanctôt, The CARESS Investigators

**Affiliations:** 1 Department of Pediatrics, McMaster University, Hamilton, Ontario Canada; 2 Department of Pediatrics, University of Calgary, Calgary, Alberta Canada; 3 Medical Outcomes and Research in Economics (MORE®) Research Group, Sunnybrook Health Sciences Centre, University of Toronto, Toronto, Ontario Canada; 4 Sunnybrook Health Sciences Centre, 2075 Bayview Avenue, Room FG-05, Toronto, Ontario M4N 3 M5 Canada

## Abstract

We examined the dosing regimens, compliance, and outcomes of premature infants who received palivizumab within the Canadian Registry of Palivizumab (CARESS). Infants receiving ≥1 dose of palivizumab during the 2006–2011 respiratory syncytial virus (RSV) seasons were recruited across 30 sites. Respiratory illness events were captured monthly. Infants ≤32 completed weeks gestational age (GA) (Group 1) were compared to 33–35 completed weeks GA infants (Group 2) following prophylaxis. In total, 6,654 patients were analyzed (Group 1, *n* = 5,183; Group 2, *n* = 1,471). The mean GA was 29.9 ± 2.9 versus 34.2 ± 2.2 weeks for Groups 1 and 2, respectively. Group differences were significant (all *p*-values <0.05) for the following: proportion of males, Caucasians, siblings, multiple births, maternal smoking, smoking during pregnancy, household smokers, >5 household individuals, birth weight, and enrolment age. Overall, infants received 92.6 % of expected injections. Group 1 received significantly more injections, but a greater proportion of Group 2 received injections within recommended intervals. The hospitalization rates were similar for Groups 1 and 2 for respiratory illness (4.7 % vs. 3.7 %, *p* = 0.1) and RSV (1.5 % vs. 1.4 %, *p* = 0.3). Neither the time to first respiratory illness [hazard ratio = 0.9, 95 % confidence interval (CI) 0.7–1.2, *p* = 0.5] nor to first RSV hospitalization (hazard ratio = 1.3, 95 % CI 0.8–2.2, *p* = 0.3) were different. Compliance with RSV prophylaxis is high. Despite the higher number of palivizumab doses in infants ≤32 completed weeks GA, the two groups’ respiratory illness and RSV-positive hospitalization rates were similar.

## Introduction

Respiratory syncytial virus (RSV) is an important viral respiratory pathogen in children in terms of individual morbidity and societal costs. The majority of children aged <2 years have experienced an RSV-related illness, predominantly during the winter months, but in some countries, RSV exposure prevails throughout the entire year.

Palivizumab is a humanized monoclonal antibody indicated for RSV prophylaxis in infants and children at high risk, and has been shown to be safe and well tolerated [1].

The Canadian Paediatric Society and most international pediatric position statements support prophylaxis for all infants of ≤32 completed weeks gestational age (GA) [2–8]. Infants of 33–35 completed weeks GA comprise a significant proportion of the annual birth cohort, and prophylaxis for this group of infants is currently based on a composite of risk factors or the use of validated risk factor models that specifically target infants at moderate to high risk for severe RSV infection and hospitalization [9–15]. In Canada, prophylaxis is recommended provincially for moderate- to high-risk 33–35 completed weeks GA infants with the use of a risk scoring tool [7, 12].

Infants of 33–35 completed weeks GA have previously been shown to have similar RSV-positive hospitalization rates and incur morbidities and costs not indifferent to those infants of ≤32 completed weeks GA [16–18]. There are limited data systematically comparing the effects of prophylaxis on the outcomes of infants in specific gestational age cohorts, following RSV-related hospitalization. The collection of long-term data on seasonality, risk factors, and outcomes is necessary in order to evaluate the impact of prophylaxis in everyday practice and examine hospitalization rates in similar premature populations globally.

The Canadian Registry of Palivizumab (CARESS) is a drug registry that prospectively collects information on patient demographics, including risk factors for RSV, palivizumab usage, characteristics of each hospitalization for both respiratory illness and RSV-positive respiratory infections, and compliance in any patient receiving palivizumab. In addition, safety data on serious adverse events possibly related to palivizumab are collected. The primary objective of the present paper is to compare data on premature infants without pre-existing medical disorders of ≤32 completed weeks GA (Group 1) to that of infants of 33–35 completed weeks GA (Group 2) within CARESS.

## Methods

Any pediatric patient receiving at least one dose of palivizumab in any RSV season from 2006 to 2011 was eligible for inclusion [19]. Children were excluded if a parent or legal guardian could not communicate in either English or French, or if the child had received palivizumab as part of a clinical trial during the study period. Only premature infants without underlying medical disorders such as bronchopulmonary dysplasia (BPD), congenital heart disease (CHD), neuromuscular impairments, cystic fibrosis (CF), dysmorphologic syndromes, chromosomal anomalies, and inherited disorders were included in this analysis. This maintained comparability of the assembled cohorts by avoiding potential confounding by known risk factors that could increase the risk of RSV hospitalization. Group 1 infants were ≤32 completed weeks GA, while Group 2 comprised infants of 33–35 completed weeks GA. Group 2 infants qualified for prophylaxis if they were considered moderate to high risk for RSV infection and hospitalization. [12].

Subjects were enrolled by the treating physician and/or research nurse. Following consent, baseline data were obtained on patient demographics, prior medical history, neonatal course, and details of current palivizumab administration. Follow-up telephone interviews were conducted monthly until the end of the RSV season, obtaining data on palivizumab administration, changes in baseline data, and specific information regarding respiratory infections.

In the event of a hospitalization, and with parental consent, the relevant hospital records were reviewed by the site’s research nurse for detailed information on the criteria for hospitalization, length of stay [regular ward vs. intensive care unit (ICU)], days on respiratory support and/or intubation, and type of RSV test performed and diagnosis, as reported in the hospital discharge summary. The study was approved by the research ethics board of each participating institution.

SPSS version 19.0 (SPSS Inc., Chicago, IL, USA) was utilized to analyze data by standard descriptive methods, comparative statistics [chi-square, analysis of variance (ANOVA), and *t*-tests] and regression analysis. Premature infants of ≤32 completed weeks GA (Group 1) were compared to infants of 33–35 completed weeks GA (Group 2). A *p*-value of less than 0.05 was considered to be significant. The primary endpoint was the time to first RSV-positive hospitalization. A Cox proportional hazards analysis was conducted to determine the effect of group on the time to first respiratory illness hospitalization and the time to first RSV-positive hospitalization, as well as to examine the effects of any potential covariates to be determined a priori.

## Results

### Subjects

Subjects were enrolled between October 2006 and May 2011. A total of 10,092 infants were recruited in 30 geographically located Canadian sites: five from Western Canada (British Columbia, Alberta, Manitoba, or Saskatchewan), 22 from Central Canada (Ontario, Quebec), and three from Eastern Canada (New Brunswick, Newfoundland, Nova Scotia, or Prince Edward Island). Of the 10,092 patients, 6,654 were included in the analysis, with 5,183 subjects categorized into Group 1 and 1,471 into Group 2. The demographic and neonatal information of the infants is displayed in Table 1. The average [± standard deviation (SD)] GA in Group 1 was 29.9 ± 2.9 weeks, and it was 34.2 ± 2.2 weeks in Group 2. There were significant differences between the groups in almost all demographic categories. Group 2 had a greater proportion of males, a greater proportion with siblings, multiple births, a mother who smokes, a mother who smoked during pregnancy, any smokers in the household, ≥2 smokers in the household, >5 individuals in the household, and, on average, the infants weighed more at birth (Table 1). Group 1 infants were more likely to be Caucasian, and, on average, were older at enrolment (Table 1). Infants in Group 1 had more complicated neonatal courses, with a greater percentage requiring respiratory support and oxygen therapy. Group 1 infants also spent a longer length of time on respiratory support and oxygen therapy. Finally, a greater proportion of Group 1 infants had documented necrotizing enterocolitis, surgery for patent ductus arteriosus, and documented sepsis. This is in keeping with their mean lower GA.

**Table 1:** Demographic and neonatal information

	Group 1 (≤32 weeks GA), *n* = 5,183	Group 2 (33–35 weeks GA), *n* = 1,471	*p*-value
Demographic information			
Mean gestational age (weeks ± SD)	29.9 ± 2.9	34.2 ± 2.2	<0.0005
No. of males (%)	2,837 (54.7)	937 (63.7)	<0.0005
No. of Caucasians (%)	3,634 (70.1)	980 (66.6)	0.011
Mean age at enrolment (months ± SD)	3.7 ± 2.9	2.3 ± 2.8	<0.0005
Mean birth weight (g ± SD)	1,430 ± 577	2,150 ± 507	<0.0005
No. in daycare (%)	48 (0.9)	33 (2.2)	<0.0005
No. with siblings (%)	2,897 (55.9)	1,146 (77.9)	<0.0005
No. of multiple births (%)	1,795 (34.6)	575 (39.1)	0.002
No. with mother that smokes (%)	720 (13.9)	242 (16.5)	0.012
No. with mother that smoked during pregnancy (%)	698 (13.5)	235 (16.0)	0.015
No. with any smokers in the household (%)	1,378 (26.6)	493 (33.5)	<0.0005
No. with ≥2 smokers at home (%)	524 (10.1)	218 (14.8)	<0.0005
No. with >5 individuals in the household (%)	1,093 (21.1)	641 (43.6)	<0.0005
No. with a history of atopy in the immediate family (%)	2,027 (39.1)	562 (38.2)	0.545
Neonatal information			
Mean length of stay (days ± SD)	54.4 ± 42.9	21.1 ± 37.4	<0.0005
No. with respiratory support (%)	3,795 (73.2)	414 (28.1)	<0.0005
Mean length of respiratory support (days ± SD)	19.3 ± 24.8	3.8 ± 5.9	<0.0005
No. with oxygen therapy (%)	3,119 (60.1)	356 (24.2)	<0.0005
Mean length of oxygen therapy (days ± SD)	26.3 ± 39.6	6.2 ± 13.4	<0.0005
No. with documented necrotizing enterocolitis (%)	215 (4.1)	15 (1.0)	<0.0005
No. with surgery for patent ductus arteriosus (%)	242 (4.7)	6 (0.4)	<0.0005
No. with documented sepsis (%)	908 (17.5)	57 (3.8)	<0.0005

### Utilization

A total of 26,540 injections were administered overall. An average of 4.0 ± 1.6 injections was given to each infant in any single RSV season. Group 1 infants, on average, received a greater number of injections in a season than Group 2 infants (4.1 ± 1.6 vs. 3.7 ± 1.5, *p* < 0.0005).

### Compliance

We defined compliance in two ways: expected number of doses versus actual number received, and inter-dose interval. For the expected number of doses, the calculation assumed monthly injections from the first dose to the end of the RSV season [11]. Overall, infants in both groups received 92.6 ± 27.9 % of the expected number of injections. Groups 1 and 2 received similar percentages of their expected injections (92.9 ± 27.9 % vs. 91.6 ± 27.9 %, *p* = 0.12).

For the number of days between injections, intervals of 30 ± 5 days were considered to be compliant. However, an interval of 20 ± 4 days between the first and second injections likely results in higher trough levels after the first dose, potentially offering better RSV protection. Therefore, an interval of 16–35 days between the first and second injections was considered to be compliant

A greater proportion of Group 2 infants received their appropriate dose-interval injections than Group 1 (75.7 % vs. 71.9 %, *p* = 0.003).

### Respiratory illness and RSV-positive hospitalizations

RSV-positive hospitalizations and associated morbidities are shown in Table 2. Apart from the mean age at the time of admission to hospital, there were no differences evident between the groups. The RSV-positive hospitalization rate was calculated follows:

$$ {\text{RSV-positive hospitalization rate}} = \frac{{\text{No. hospitalized for respiratory illness}}}{{\text{Total no. of children}}} \times \frac{{\text{No. of RSV-positive children}}}{{\text{Total no. of children tested}}} $$

**Table 2:** Respiratory syncytial virus (RSV)-positive hospitalizations

	Group 1 (≤32 weeks GA), *n* = 62	Group 2 (33–35 weeks GA), *n* = 18	*p*-value
Mean age at admission (months ± SD)	4.9 ± 2.8	2.8 ± 2.5	0.004
Mean length of stay (days ± SD)	6.7 ± 5.4	5.2 ± 5.0	0.276
No. requiring intubation (%)	4 (6.5)	1 (5.6)	1.000
Mean length of intubation (days ± SD)	0.5 ± 2.2	0.2 ± 0.9	0.556
No. requiring respiratory support (%)	14 (22.6)	4 (22.2)	1.000
Mean length of respiratory support (days ± SD)	1.3 ± 2.9	0.3 ± 1.8	0.184
No. admitted to ICU (%)	16 (25.8)	5 (27.8)	1.000
Mean length of ICU stay (days ± SD)	1.2 ± 2.7	1.3 ± 2.4	0.906

**Fig. 1:**
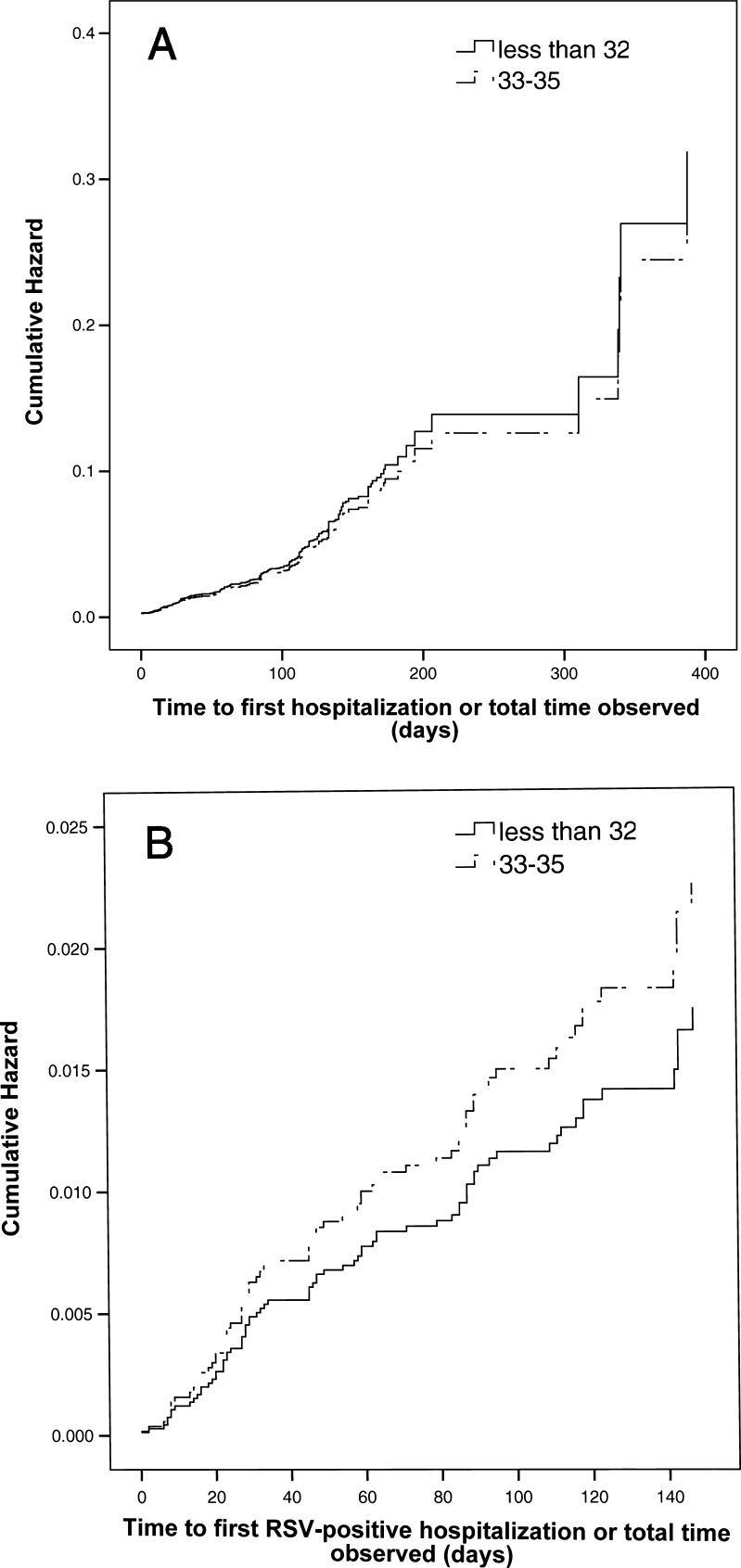
Cox proportional hazard curves depicting the effect of group allocation on the time to hospitalization for respiratory illness (**a**) and respiratory syncytial virus (RSV)-positive infection (**b**), comparing infants of ≤32 completed weeks gestational age (GA; *solid lines*) and those of 33–35 completed weeks GA (*dotted lines*). Group allocation did not have an effect in either case [respiratory illness: hazard ratio = 0.9, 95 % confidence interval (CI) 0.70–1.2, *p* = 0.5; RSV-positive infection: hazard ratio = 1.3, 95 % CI 0.8–2.2, *p* = 0.3]

Overall, 297 infants were hospitalized 339 times for respiratory illnesses, giving a hospitalization incidence rate of 4.5 %. Of these, 243 were tested for RSV, and 80 were positive, giving an overall RSV-positive hospitalization rate of 1.47 %. Groups 1 and 2 did not have significantly different hospitalization rates for respiratory-related events (4.7 % vs. 3.7 %, *p* = 0.1) nor RSV-positive infections (1.5 % vs. 1.4 %, *p* = 0.3). Group allocation (Group 1 vs. Group 2) also did not have a significant effect on the time to first hospitalization [hazard ratio = 0.9, 95 % confidence interval (CI) 0.7–1.2, *p* = 0.5] nor on the time to first RSV-positive hospitalization (hazard ratio = 1.3, 95 % CI 0.8–2.2, *p* = 0.3) (Fig. 1).

**Fig. 2:**
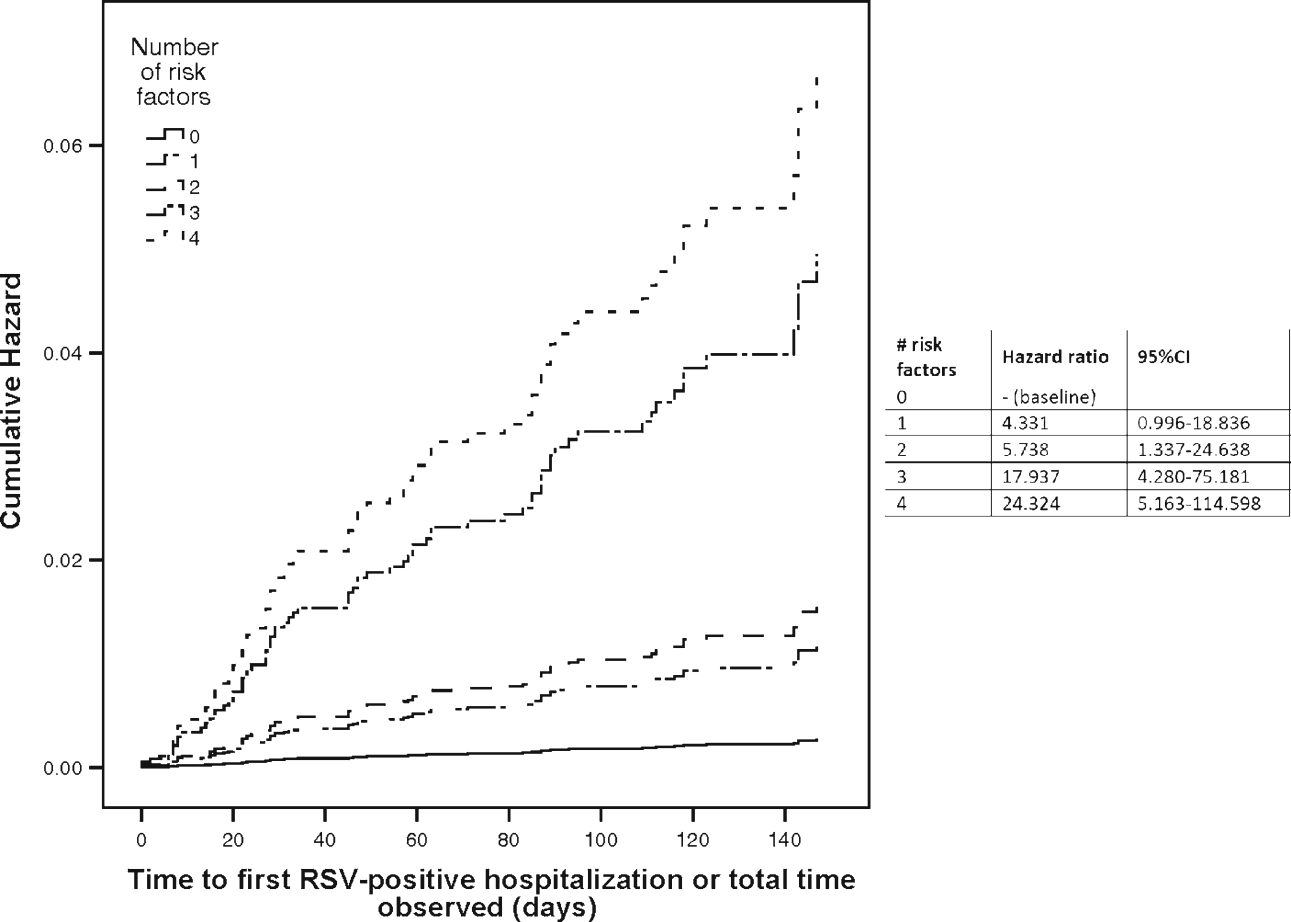
Cox proportional hazard curves illustrating the number of risk factors that had a significant effect on the time to first RSV-positive hospitalization: having siblings, >5 individuals in the household, a history of atopy in the immediate family, and smoking in the household

Table 3 describes the demographic differences between infants who were hospitalized with an RSV infection and those who were either not hospitalized or who had a non-RSV-positive hospitalization. A greater proportion of hospitalized infants had siblings, a mother who smokes, smokers at home, >5 individuals in the household, and a history of atopy in the immediate family. A Cox proportional hazards analysis found that each of these risk factors independently had a significant effect on the time to first RSV-positive hospitalization (siblings: hazard ratio = 2.5, 95 % CI 1.3–4.8, *p* = 0.004; >5 individuals in the household: hazard ratio = 2.1, 95 % CI 1.8–3.3, *p* = 0.003; history of atopy: hazard ratio = 2.3, 95 % CI 1.4–3.7, *p* = 0.001; smokers: hazard ratio = 1.7, 95 % CI 1.0–2.6, *p* = 0.035). Interestingly, cumulative risk factors in an individual infant significantly increased the hazard ratio (Fig. 2), from 4.3 (95 % CI 1.0–18.8) with one risk factor to 24.3 (95 % CI 5.1–114.6) with four risk factors. However, the Cox proportional hazards analysis showed no effect of group allocation (*p* = 0.640), even when the number of risk factors are taken into consideration (model: chi-square = 47.21, df = 2, *p* < 0.0005).

**Table 3:** Demographic and neonatal information for patients who were and were not hospitalized for an RSV infection

	RSV-positive hospitalization, *n* = 80	Non-RSV-positive hospitalization or not hospitalized, *n* = 6,574	*p*-value
Demographic information			
Mean gestational age (weeks ± SD)	30.5 ± 3.2	30.9 ± 3.3	0.260
No. of males (%)	42 (52.5)	3,732 (56.8)	0.429
No. of Caucasians (%)	60 (75.0)	4,554 (69.3)	0.329
Mean age at enrolment (months ± SD)	3.0 ± 2.0	3.4 ± 2.9	0.259
Mean birth weight (g ± SD)	1,598 ± 957	1,589 ± 631	0.901
No. in daycare (%)	0 (0)	81 (1.2)	0.628
No. with siblings (%)	66 (82.5)	3,977 (60.5)	<0.0005
No. of multiple births (%)	34 (42.5)	2,336 (35.5)	0.198
No. with mother that smokes (%)	19 (23.8)	944 (14.4)	0.023
No. with mother that smoked during pregnancy (%)	17 (21.3)	916 (13.9)	0.074
No. with smokers in the household (%)	32 (40.0)	1,839 (28.0)	0.024
No. with ≥2 smokers at home (%)	13 (16.3)	729 (11.1)	0.151
No. with >5 individuals in the household (%)	35 (43.8)	1,699 (25.8)	0.001
No. with a history of atopy in the immediate family (%)	48 (60.0)	2,541 (38.7)	<0.0005
Neonatal information			
Mean length of stay (days ± SD)	54.6 ± 46.9	46.7 ± 44.0	0.128
No. with respiratory support (%)	54 (67.5)	4,155 (63.2)	0.485
Mean length of respiratory support (days ± SD)	25.3 ± 27.4	17.7 ± 24.0	0.022
No. with oxygen therapy (%)	49 (61.3)	3,426 (52.1)	0.115
Mean length of oxygen therapy (days ± SD)	28.3 ± 35.1	24.2 ± 38.3	0.462
No. with documented necrotizing enterocolitis (%)	4 (5.0)	226 (3.4)	0.358
No. with surgery for patent ductus arteriosus (%)	8 (10.0)	240 (3.7)	0.010
No. with documented sepsis (%)	14 (17.5)	951 (14.5)	0.425

## Discussion

RSV-related hospitalization rates for premature infants of ≤35 weeks GA range from 11 to 28 % [20–25] and are not indifferent across the gestational age group strata. Boyce et al., in a retrospective Tennessee Medicaid study, reported that infants of <28 weeks, 29–32 weeks, and 33–35 weeks GA compared to healthy term enrollees had RSV hospitalizations per 1,000 child years of the RSV season of 187.5, 163.6, and 159.6 versus 88.2, respectively [21]. Similarly, Heikkinen et al. determined RSV hospitalizations in preterm infants <2 years of age from 1991–2000 and documented an incidence of 7 % in those who were <28 weeks GA (*n* = 168), 7 % in 29–32 weeks GA (*n* = 498), and 4 % in premature infants 33–35 weeks GA (*n* = 1,133) [26]. Apnea may be a marker of RSV disease severity in young infants [27, 28], but the risk could not be quantified in a systematic review [29]. Schiller et al. noted that, of 31 preterm infants admitted to the pediatric ICU between 2004 and 2007, the incidence of central apnea ranged from 3.2 % (<28 weeks GA) to 32.3 % (28–32 weeks GA) and 64.5 % (33–36 weeks GA) [30]. Additionally, data on morbidities and outcomes in 33–35 weeks GA infants following an RSV illness indicate that they are similar or worse than those born at ≤32 weeks in terms of rates of apnea, intubation, ventilation, oxygen supplementation, systemic complications, ICU admission, hospital and ICU length of stay, and median costs for patient care [16–18]. In a matched-control study of 32–35 weeks GA infants hospitalized with RSV, follow-up healthcare resource utilization up to a mean of 2.1 years of age was significantly higher in index cases, with a higher incidence of overall accompanying mortality and sudden or unexplained death [31]. Following hospitalization of 32–35 weeks GA infants with RSV, parents’ health-related quality of life is also significantly impacted, with more and longer time off work for child care, excess workload related to infant illness, and burden associated with lower scores in physical activities of daily living [32]. Fatalities for hospitalized premature infants across 36 published studies (1966–2009) are between 0 and 6.1 % [33] and low birth weight <2,500 g is an independent risk factor for mortality from bronchiolitis [34].

The safety and efficacy of RSV prophylaxis in the reduction of RSV hospitalizations has been demonstrated in a randomized, double-blind, clinical trial, and is more significant for infants of 32–35 weeks GA without BPD (80 % reduction over placebo, *p* = 0.002) compared to infants of <32 weeks GA (47 % reduction over placebo, *p* = 0.003) [1]. While the use of prophylaxis has been adopted almost universally for infants of <32 weeks GA, there has been a guarded approach to RSV immunization in the 33–35 weeks GA cohort, despite the supportive scientific evidence. This is not surprising, because palivizumab is costly and this group comprises a significant proportion of annual births which has steadily climbed over recent years universally, given the increasing number of deliveries of late preterm infants. However, the use of specific risk factors weighted against the risk of RSV-positive hospitalization [10, 11] or validated risk models based on defined variables has streamlined a more conservative, cost-effective prophylaxis program for 33–35 weeks GA infants [12, 13, 35–37].

In our study, RSV-positive hospitalization rates for infants of both ≤32 and 33–35 weeks GA following prophylaxis were similar at 1.5 and 1.4 %, respectively, and were lower than similar groups in the treated arm of the IMpact trial (5.8 and 1.8 %, respectively), despite a three-fold greater sample size [1]. This real-world experience of the improved effectiveness of palivizumab was also noted in the United States Outcomes Registry, which realized higher hospitalization rates of 1.84 % for infants of <32 weeks GA (*n* = 7,786) and lower rates of 0.83 % for infants of 32–35 weeks GA (*n* = 9,294), respectively, with significantly higher numbers of recruited infants who had similar gestational ages and pre-existing medical disorders such as CHD, BPD, and CF [38]. The risk scoring tool, or a modified version thereof, is used in all of the Canadian provinces to target RSV prophylaxis cost-effectively for 33–35 weeks GA infants who are considered to be at moderate to high risk (risk score 49–100) for RSV hospitalization [12]. This may account for the difference in RSV-positive hospitalization rates compared to the United States Outcomes Registry [38]. Of interest, the overall compliance in our study relative to the expected number of palivizumab doses and 30 ± 5 day dose intervals during the RSV season was higher at 92.6 % ± 27.9 % versus 79.9 to 82.7 % and 71.9 to 75.7 % versus 65.2 to 69.5 %, respectively, in the United States Outcomes Registry, which may further explain some of the differences in the RSV-related hospitalization rates [38].

Several environmental and demographic risk factors are associated with severe RSV infection and subsequent hospitalization. These invariably include prematurity, sex, household crowding, daycare attendance, exposure to tobacco smoke and maternal smoking during pregnancy, young siblings, chronological age and birth early during the RSV season, intrauterine growth restriction, familial atopy and asthma, and short duration of breast feeding [4, 10, 11, 13, 39–45]. We established that, in premature infants of <35 weeks GA who had received palivizumab, similar risk factors such as having siblings, >5 individuals in the household, a history of atopy in the immediate family, and smoking in the household also determined the time to first RSV hospitalization. The effect of each risk factor was independently significant and was augmented six-fold as the number of risk factors cumulatively rose from 1 to 4 in an individual patient. Our study confirms the relationship between environmental smoke exposure and RSV lower respiratory tract infection hospitalization, which is similar to other documented reports, but the effect is not uniformly consistent across published studies [46–48] because the association is likely related to the dose and concentration of circulating cigarette smoke extract [49, 50].

The limitations of our study include the absence of control groups for infants who received prophylaxis, which affected our ability to interpret the magnitude of the reduction in RSV hospitalization, apart from comparisons with historical controls from the IMpact study [1]. Subjects of 33–35 weeks GA and considered to be low risk (score 0–48) are not enrolled in the CARESS database and, therefore, we cannot draw any conclusions on the outcomes of this cohort. However, in a previous study involving 430 infants of 33–35 weeks GA, the low-risk group comprised 81 % of the patients, of whom only 1.4 % were hospitalized with an RSV infection [51].

In summary, this is the largest study that determined the outcomes of premature infants of ≤35 completed weeks GA without underlying medical disorders who received prophylaxis in a single RSV season over four consecutive years. RSV hospitalizations were similar for infants of ≤32 weeks GA and those between 33 and 35 weeks GA, despite the greater number of doses received by infants of ≤32 weeks GA. The low hospitalization rate could be, in part, related to the high compliance achieved with infants receiving 92.6 % of their expected number of injections and the higher efficacy of palivizumab, as demonstrated in the original randomized controlled trial [1]. RSV prophylaxis of infants of 33–35 weeks GA is both beneficial and cost-effective, but should be rationalized based on country-specific epidemiological data, local financial budgets and funding availability, and should be strategized to target the highest risk infants in this sub-population.
